# Inactivation of presenilins causes pre-synaptic impairment prior to post-synaptic dysfunction

**DOI:** 10.1111/j.1471-4159.2010.07011.x

**Published:** 2010-12

**Authors:** Dawei Zhang, Chen Zhang, Angela Ho, Alfredo Kirkwood, Thomas C Südhof, Jie Shen

**Affiliations:** 1 Center for Neurologic Diseases, Brigham and Women’s Hospital, Program in Neuroscience, Harvard Medical SchoolBoston, Massachusetts, USA; 2 Department of Molecular and Cellular Physiology, Howard Hughes Medical Institute, Stanford University School of MedicinePalo Alto, California, USA; 3 Department of Biology, Boston UniversityBoston, Massachusetts, USA; 4 Mind/Brain Institute, Johns Hopkins UniversityBaltimore, Maryland, USA

**Keywords:** Alzheimer’s disease, conditional knockout, LTP, neurodegeneration, neurotransmitter release, NMDA receptor, synaptic dysfunction, synaptic facilitation

## Abstract

**Abstract:**

Synaptic dysfunction is widely thought to be a pathogenic precursor to neurodegeneration in Alzheimer’s disease (AD), and the extent of synaptic loss provides the best correlate for the severity of dementia in AD patients. Presenilins 1 and 2 are the major causative genes of early-onset familial AD. Conditional inactivation of presenilins in the adult cerebral cortex results in synaptic dysfunction and memory impairment, followed by age-dependent neurodegeneration. To characterize further the consequence of presenilin inactivation in the synapse, we evaluated the temporal development of pre-synaptic and post-synaptic deficits in the Schaeffer-collateral pathway of *presenilin* conditional double knockout (*PS* cDKO) mice prior to onset of neurodegeneration. Following presenilin inactivation at 4 weeks, synaptic facilitation and probability of neurotransmitter release are impaired in *PS* cDKO mice at 5 weeks of age, whereas post-synaptic NMDA receptor (NMDAR)-mediated responses are normal at 5 weeks but impaired at 6 weeks of age. Long-term potentiation induced by theta burst stimulation is also reduced in *PS* cDKO mice at 6 weeks of age. These results show that loss of presenilins results in pre-synaptic deficits in short-term plasticity and probability of neurotransmitter release prior to post-synaptic NMDAR dysfunction, raising the possibility that presenilins may regulate post-synaptic NMDAR function in part *via* a trans-synaptic mechanism.

Alzheimer’s disease (AD) is characterized clinically by progressive memory loss and other cognitive disabilities, and neuropathologically by synaptic and neuronal loss as well as accumulation of extracellular amyloid plaques and intraneuronal fibrillary tangles. The *presenilin* genes harbor the majority of the mutations linked to familial forms of early-onset AD (Levy-Lahad *et al.* 1995; Rogaev *et al.* 1995; Sherrington *et al.* 1995). Presenilin, along with Aph1, Pen2 and Nicastrin, are integral components of a multiprotein protease complex, termed γ-secretase, which is responsible for the intramembranous cleavage of type I transmembrane proteins, such as the amyloid precursor protein and Notch receptors (De Strooper *et al.* 1998, 1999; Fortini 2002). During development, presenilin plays a major role in the maintenance of neural progenitor population through the regulation of the Notch signaling pathway (Shen *et al.* 1997; Handler *et al.* 2000; Wines-Samuelson and Shen 2005; Wines-Samuelson *et al.* 2005; Kim and Shen 2008). In the adult brain, presenilin is expressed highly in excitatory neurons of the cerebral cortex, and is required for memory formation and age-dependent neuronal survival in a γ-secretase-dependent manner (Yu *et al.* 2001; Feng *et al.* 2004; Saura *et al.* 2004; Tabuchi *et al.* 2009; Wines-Samuelson *et al.* 2010).

Synaptic dysfunction is widely thought to be a key pathogenic event before frank neuronal death in AD pathogenesis (Hsia *et al.* 1999; Saura *et al.* 2004). Inactivation of presenilin in conditional double knockout (*PS* cDKO) results in striking pre- and post-synaptic impairments prior to progressive neurodegeneration that resemble key neuropathological features of AD, raising the possibility that partial loss of presenilin function may contribute to synaptic dysfunction and neurodegeneration in AD (Saura *et al.* 2004; Shen and Kelleher 2007; Zhang *et al.* 2009; Wines-Samuelson *et al.* 2010). Further dissection of synaptic dysfunction caused by loss of presenilin may provide additional insight into the mechanisms underlying age-related synaptic loss and neuronal degeneration in AD.

In the current study, we performed a detailed electrophysiological analysis in the hippocampal Schaeffer-collateral pathway to establish temporal development of the pre- and post-synaptic defects caused by loss of presenilin in *PS* cDKO mice. We found that the pre-synaptic deficits, such as frequency facilitation and Ca^2+^ dependency of facilitation, occur prior to post-synaptic NMDA receptor (NMDAR) deficits. Furthermore, we show that the pre-synaptic deficits are associated with a reduction in release probability. Our study suggests that pre-synaptic deficit in neurotransmitter release is the initial impairment prior to post-synaptic NMDAR-mediated dysfunction in *PS* cDKO mice.

## Materials and methods

### Mice

The generation of *PS* cDKO mice has been previously described (Saura *et al.* 2004). Briefly, f*PS1*/f*PS1;PS2*^*−/−*^*;CamKII-Cre* mice were bred with *fPS1/fPS1;PS2*^*−/−*^ mice to obtain *PS* cDKO mice (*fPS1/fPS1;PS2*^*−/−*^*;Cre*) and *fPS1/fPS1;Cre* were bred with *fPS1/fPS1* to obtain control mice (*fPS1/fPS1*). The genetic background of the *PS* cDKO and control mice was similar in the C57BL6/129 hybrid background.

### Western analysis

Quantitative western blots were carried out as previously described (Ho *et al.* 2006). Briefly, equal amount of protein were resolved on sodium dodecyl sulfate–polyacrylamide gel electrophoresis and radioisotope ^125^I-labeled secondary antibodies were used for quantitative analyses followed by PhosphorImager (Molecular Dynamics, Sunnyvale, CA, USA).

### Electrophysiological analysis

Electrophysiology experiments were performed on male *PS* cDKO mice at various ages. Acute hippocampal slices at 400 μm were prepared using a vibratome 2000 as previously described (Saura *et al.* 2004). Slices were maintained in a storage chamber containing oxygenized artificial CSF (aCSF in mM: 124 NaCl, 5 KCl, 1.25 NaH_2_PO_4_, 1.3 MgCl_2_, 2.6 CaCl_2_, 26 NaHCO_3_, 10 glucose, pH 7.4, 310–315 mOsm) at 25°C after 1-h recovery at 30°C. Synaptic responses were evoked at the Schaeffer-collateral pathway by a bipolar concentric metal electrode (500-μs biphasic pulses), and recorded with aCSF-filled glass microelectrodes (1–2 MΩ) extracellularly in CA1 stratum radiatum. The recording chamber and aCSF perfusion solution was kept at 30°C. Long-term potentiation (LTP) was induced by five episodes of theta burst stimulation (TBS) delivered at 0.1 Hz. Each episode contains ten stimulus trains (five pulses at 100 Hz) delivered at 5 Hz. Average responses (mean ± SEM) are expressed as percentage of pre-TBS baseline responses, which were evoked by half-maximum stimulation intensity with 0.2 ms pulses at 0.033 Hz. NMDAR responses were recorded in Mg^2+^-free aCSF containing 10 μM 6-cyano-7-nitroquinoxaline-2,3-dione (CNQX) and 100 μM picrotoxin, which block α-amino-3-hydroxy-5-methylisoxazole-4-propionate (AMPA) receptor- and GABA type A receptor-mediated responses, respectively. Whole-cell recordings were performed in CA1 pyramidal neurons visually identified with an IR-DIC BX51 Olympus microscope (Olympus Corporation, Tokyo, Japan). Patch pipettes (2–4 MΩ) were filled with internal solution consisting of (in mM): 130 CsCl, 8 KCl, 10 EGTA, 10 HEPES, 5 QX-314, 3 ATP, 0.3 Na_2_GTP, pH 7.2, 280–285 mOsm. Chemicals were from Sigma unless otherwise noted. Experimenters were blind to the genotypes of the mice.

### Statistics

All data shown are mean ± SEM. Statistical analysis was performed with Student’s *t*-test, *p < 0.05; **p < 0.01; ***p < 0.001.

## Results

### Impaired frequency facilitation in *PS* cDKO mice at 5 weeks of age

**Fig 1:**
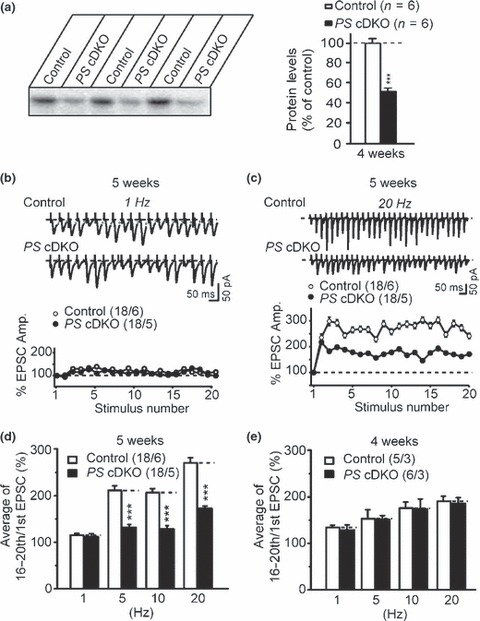
Impaired frequency facilitation in *PS* cDKO mice. (a) Reduction of PS1 in cortical lysates of *PS* cDKO mice at 4 weeks of age. (b–c) Representative traces from 5-week-old control and *PS* cDKO mice at 1 and 20 Hz stimulation frequency, respectively. Averaged responses to stimulus trains of 1 and 20 Hz stimulations recorded from control and *PS* cDKO mice. Values of EPSC amplitude were normalized to the amplitude of first EPSC in stimulation trains. (d) Summary graph comparing frequency dependence of synaptic facilitation in 5-week-old *PS* cDKO mice. (e) Summary graph comparing frequency dependence of synaptic facilitation in 4-week-old *PS* cDKO mice. The values in parentheses indicate the number of hippocampal neurons (left) and the number of mice (right) used in each experiment. ***p < 0.001.

Our previous western analysis showed that PS1 expression begins to be reduced in the cerebral cortex of *PS* cDKO mice between postnatal days 18 and 22 (Yu *et al.* 2001). At 2 months of age, in addition to spatial and associative memory impairments, marked pre- and post-synaptic defects, including paired-pulse facilitation and NMDAR-mediated responses, are observed in the hippocampal Schaeffer-collateral pathway of *PS* cDKO mice (Saura *et al.* 2004). To establish the time course of temporal development of these pre-synaptic and post-synaptic defects, we evaluated pre-synaptic and post-synaptic function in *PS* cDKO mice before the age of 2 months. Our western analysis showed ∼ 50% reduction of PS1 in the cerebral cortex of *PS* cDKO mice at 4 weeks of age (Fig. 1a). The residual PS1 in cortical lysates prepared from *PS* cDKO mice is because of expression of PS1 in glia, interneurons and perhaps a small percentage of excitatory neurons that lack Cre expression (Yu *et al.* 2001).

To establish the temporal development of pre-synaptic deficits in *PS* cDKO mice, we analyzed frequency-dependent synaptic facilitation, one form of short-term plasticity that is dependent primarily on pre-synaptic release probability (Pr) at CA3–CA1 hippocampal synapses (Zucker and Regehr 2002). Excitatory post-synaptic currents (EPSCs) were evoked by extracellular stimulation of Schaeffer-collateral commissural fibers. We recorded EPSCs evoked by stimulus trains of 20 action potentials at frequencies ranging from 1 to 20 Hz in CA1 pyramidal neurons of acute hippocampal slices. At 4 weeks of age, the EPSC amplitude of synaptic facilitation at all four frequencies was normal in *PS* cDKO mice (Fig. 1e). By 5 week of age, we found that synaptic facilitation of EPSC amplitude was significantly reduced at stimulation frequencies higher than 5 Hz in *PS* cDKO mice, whereas synaptic facilitation elicited at 1 Hz stimulus was not changed (Fig. 1b–d). Similar results were obtained by measurement of frequency-dependent facilitation of excitatory post-synaptic potential (EPSP) slope using extracellular recording of the Schaeffer-collateral pathway of *PS* cDKO mice (data not shown). These results show that frequency facilitation impairments begin at 5 weeks of age in *PS* cDKO mice.

### Calcium-dependency of synaptic facilitation

**Fig 2:**
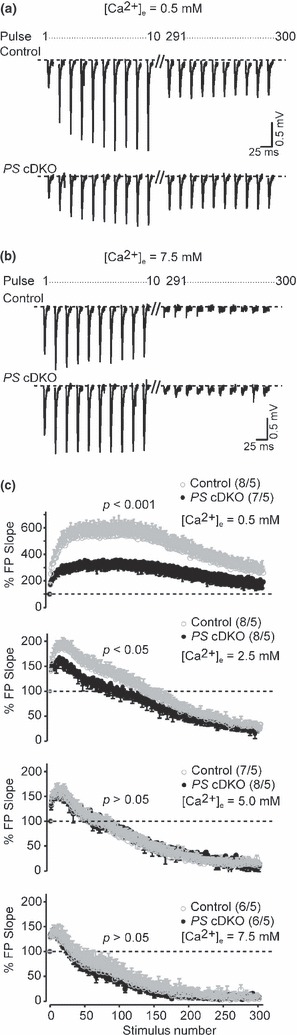
Calcium dependency of synaptic facilitation in *PS* cDKO mice. (a–b) Representative traces of synaptic facilitation from 5-week-old control and *PS* cDKO mice at external calcium concentration ([Ca^2+^]_e_ = 0.5 and 7.5 mM, respectively). (c) Synaptic responses were elicited by high frequency stimulation at different extracellular calcium concentration ([Ca^2+^_e_ = 0.5, 2.5, 5.0 and 7.5 mM) between control and *PS* cDKO mice. Values of EPSP slope were normalized to the first stimulus in a stimulation train. The values in parentheses indicate the number of hippocampal slices (left) and the number of mice (right) used in each experiment.

To examine whether the deficit in synaptic facilitation in *PS* cDKO mice at 5 weeks of age is calcium-dependent and can be rescued by higher external Ca^2+^ concentration, we measured calcium-dependence of synaptic facilitation by changing external calcium concentration. Synaptic responses were elicited by stimulus trains containing 300 pulses at 14 Hz. In *PS* cDKO mice, frequency facilitation deficits were calcium-dependent. Synaptic facilitation was significantly reduced at low external calcium concentration ([Ca^2+^]_e_ = 0.5 mM) in *PS* cDKO mice (Fig. 2a and c). When [Ca^2+^]_e_ was increased to 2.5 mM, synaptic facilitation was still reduced; however, the difference is much smaller (Fig. 2c). Conversely, synaptic facilitation is indistinguishable at 5 and 7.5 mM external calcium concentration (Fig. 2b and c). We also measured calcium dependency in 4-week-old *PS* cDKO mice, and found no difference compared with control mice (data not shown), which is consistent with the observation that frequency facilitation underlying physiological calcium concentration is normal at 4 weeks of age, but impaired at 5 weeks of age in *PS* cDKO mice (Fig. 1). Interestingly, at 2.5 mM external calcium concentration, the decrease of synaptic responses underlying 14 Hz stimulus trains (300 pulses), which is thought to reflect the rate of depletion of releasable vesicles pool, were not affected in 5-week-old *PS* cDKO mice (averages of last 20 EPSCs in 300-pulse trains were compared; control = 28.8 ± 5.3%; *PS* cDKO = 31.1 ± 4.4%), arguing against an impairment of synaptic vesicle recycling as a cause of the decreased synaptic facilitation. These results demonstrate that the frequency facilitation deficit caused by presenilin inactivation is calcium-dependent.

### Reduced release probability in *PS* cDKO mice

**Fig 3:**
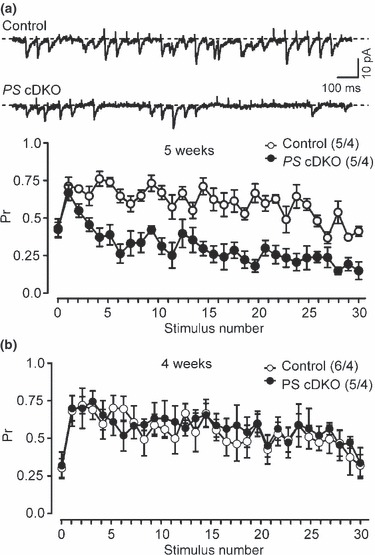
Reduced release probability (Pr) in *PS* cDKO mice. (a) Representative traces of unitary EPSCs recorded in response to short trains from control and *PS* cDKO mice at 5 weeks of age. Plot of Pr during repetitive stimulation showing reduced Pr at unitary synapses from control and *PS* cDKO mice. (b) Normal Pr during repetitive stimulation in *PS* cDKO mice at 4 weeks of age. The values in parentheses indicate the number of hippocampal neurons (left) and the number of mice (right) used in each experiment.

The calcium-dependence frequency facilitation deficit caused by presenilin inactivation at 5 weeks of age suggests that probability of release (Pr) during repetitive stimulation is reduced in *PS* cDKO mice. To test this directly, we examined Pr by recording trains of unitary synaptic responses evoked by low intensity stimulation. The plot of occurrence of unitary responses directly reflects the release probability at the single recording synapse. Probability of release during 20 Hz stimulus trains was normal in *PS* cDKO mice at 4 weeks of age (Fig. 3b), but it was significantly reduced at 5 weeks of age (Fig. 3a). These results show that probability of neurotransmitter release is impaired in *PS* cDKO mice at 5 weeks of age.

### Reduced NMDAR-mediated responses in *PS* cDKO mice

**Fig 4:**
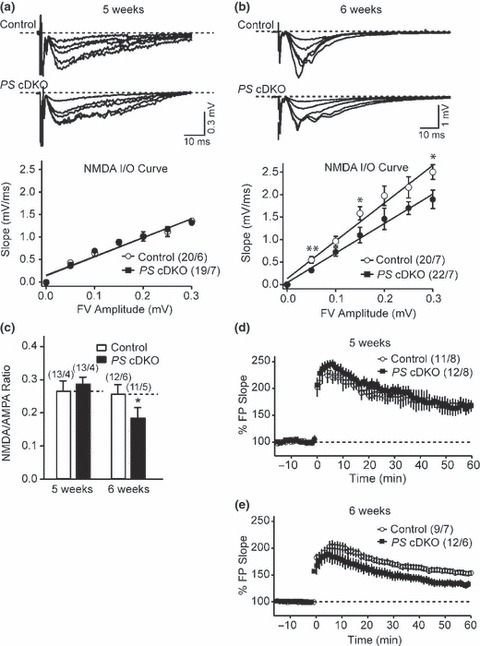
Decreased NMDAR-mediated responses in *PS* cDKO mice. (a) Normal input/output (I/O) curve for NMDAR-mediated responses in *PS* cDKO mice at 5 weeks of age. The initial slope of NMDAR-mediated synaptic responses was plotted as a function of fiber volley (FV). (b) Reduced NMDAR-mediated I/O curve in *PS* cDKO mice at 6 weeks of age. (c) Reduced NMDAR/AMPAR ratio in *PS* cDKO mice at 6 weeks of age but not at 5 weeks of age. (d) Reduced TBS-induced LTP in *PS* cDKO mice at 6 weeks of age but not at 5 weeks of age. The values in parentheses indicate the number of hippocampal slices (left) and the number of mice (right) used in each experiment. *p < 0.05 and **p < 0.01.

We next examined the temporal development of post-synaptic deficits in *PS* cDKO mice by measuring NMDAR-mediated synaptic responses at 5 weeks of age, when pre-synaptic changes in short-term plasticity were observed (Figs 1–3). NMDAR-mediated synaptic responses were measured by pharmacologically applying blockers of AMPA and GABA_A_ receptors (10 μM CNQX and 100 μM picrotoxin, respectively). We found that input-output curve of NMDAR-mediated synaptic responses, is normal in *PS* cDKO mice at 5 weeks of age (Fig. 4a). However, at 6 weeks of age, we observed a small but significant reduction in NMDAR-dependent responses in *PS* cDKO (Fig. 4b). Furthermore, we quantified the ratio of NMDAR- to AMPA receptor-mediated responses recorded from CA1 pyramidal neurons, a more precise measurement in monitoring post-synaptic NMDARs. AMPA receptor-mediated response was recorded at −70 mV holding potential in the presence of Mg^2+^ to block NMDAR-mediated component. NMDAR-mediated response was measured 75 ms after the peak of synaptic responses recorded at +40 mV holding potential. Consistently, we found similar temporal patterns in NMDA/AMPA ratio: normal at 5 weeks and impaired at 6 weeks of age in *PS* cDKO mice (Fig. 4c).

Next, we measured LTP in hippocampal Schaffer-collateral pathway of *PS* cDKO mice. LTP was induced by five trains of TBS and the magnitude of LTP measured 60 min after induction was indistinguishable in *PS* cDKO and control mice at 5 weeks of age (Fig. 4d). However, LTP was significantly impaired in *PS* cDKO mice at 6 weeks of age (Fig. 4d). Together, these results show that *PS* cDKO mice exhibit deficits in synaptic transmission (both short and long-term plasticity) and pre-synaptic changes in neurotransmitter release occur prior to the post-synaptic receptor changes in *PS* cDKO mice.

## Discussion

It is well known that the extent of synaptic loss provides the best correlates to the severity of cognitive impairments during the early and late stages of AD (Hanks and Flood 1991; Terry *et al.* 1991; Braak and Braak 1997). Loss of presenilin function and accumulation of β-amyloid peptides can induce synaptic impairment in the absence of neuronal degeneration (Hsia *et al.* 1999; Saura *et al.* 2004; Zhang *et al.* 2009), suggesting that synaptic dysfunction may be a pathogenic precursor before frank neurodegeneration in AD. Our prior analysis of *PS* cDKO mice at 2 months of age, before the onset of neurodegeneration, revealed that loss of presenilin function in the adult cerebral cortex causes both pre-synaptic and post-synaptic deficits, such as paired pulse facilitation, NMDAR-mediated responses and LTP (Saura *et al.* 2004). In the current study, we pursued further the role of presenilins in the synapse by exploring the temporal development of pre-synaptic and post-synaptic deficits in the CA3–CA1 synapse of *PS* cDKO mice, in which presenilins are inactivated in both CA3 and CA1 hippocampal neurons at 4 weeks of age. We found that impaired synaptic function in *PS* cDKO mice is age-dependent and the earliest synaptic changes occur at 5 weeks of age, when pre-synaptic deficit in neurotransmitter release is the initial impairment prior to post-synaptic dysfunction. We found that frequency facilitations are selectively impaired in a calcium-dependent manner, whereas use-dependent depression is normal in *PS* cDKO mice. Furthermore, probability of evoked glutamate release during repetitive stimulus trains is also substantially reduced, providing further evidence for pre-synaptic deficits in short-term plasticity. In contrast, post-synaptic NMDAR-mediated responses are normal at 5 weeks of age in *PS* cDKO mice. It is not until 6 weeks of age, we observed reduced post-synaptic NMDAR-mediated responses in *PS* cDKO mice. Our studies suggest that the pre-synaptic deficits occur prior to post-synaptic NMDAR dysfunction in the absence of presenilins at mature synapses, raising the possibility that post-synaptic NMDAR alteration might be caused in part *via* a trans-synaptic mechanism.

**Fig 5:**
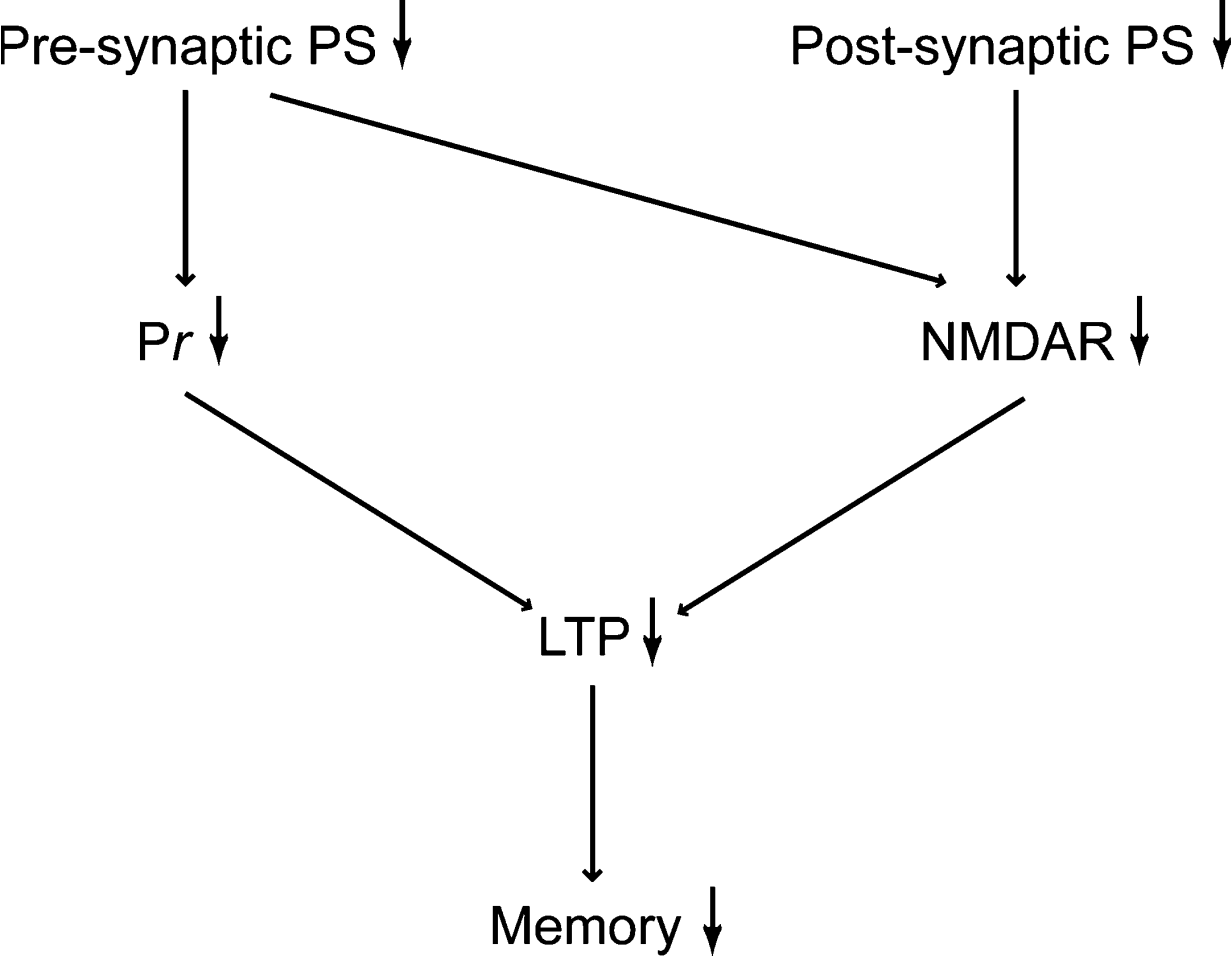
A model depicts how presenilins regulate synaptic function and memory. Presenilins can promote LTP induction and memory *via* two independent mechanisms: pre-synaptic presenilin regulates probability of glutamate release through its control of calcium release from the ER, whereas both pre-synaptic and post-synaptic presenilins are involved in the regulation of NMDAR functions.

Notably, our data showed that synaptic facilitation is impaired in a calcium-dependent manner in *PS* cDKO mice, which support the notion that inactivation of presenilins affects calcium homeostasis and induces pre-synaptic deficits. Our recent report using two independent lines of more restricted *PS* cDKO mice, in which presenilins are inactivated in either CA3 or CA1 neurons, demonstrated that loss of pre-synaptic presenilins alone is sufficient to cause defects in glutamate release and LTP *via* alterations in intracellular calcium signaling (Zhang *et al.* 2009). However, inactivation of presenilins in either pre-synaptic CA3 or post-synaptic CA1 neurons alone is insufficient to cause NMDAR deficits, suggesting that both cell-autonomous and trans-synaptic mechanisms are at work in presenilin-dependent NMDAR functions (Fig. 5). The findings from the current study showing NMDAR impairment follows pre-synaptic deficits in *PS* cDKO mice lacking presenilins in both CA3 and CA1 neurons provide further support for this trans-synaptic mechanism. Thus, LTP deficits in the absence of presenilins are likely caused by both the neurotransmitter release impairment because of loss of pre-synaptic presenilins and the NMDAR dysfunction because of loss of pre-synaptic and post-synaptic presenilins (Fig. 5). These findings raised the possibility that impaired pre-synaptic glutamate release and NMDAR function may both play roles in AD pathophysiology.

Impairment in neurotransmitter release may be one of the earliest pathogenic changes prior to neurodegeneration in AD. Recent studies show that PINK1, DJ-1 and Parkin, proteins associated with familial Parkinson’s disease, are crucial for action-potential induced dopamine release from nigrostriatal terminals and striatal synaptic plasticity without detectable neurodegeneration (Goldberg *et al.* 2005; Kitada *et al.* 2007, 2009). Together with our observations, it seems possible that reduced neurotransmitter release may represent a convergent mechanism leading to neurodegeneration in affected circuits in AD and Parkinson’s disease, and perhaps other neurodegenerative diseases.

## Abbreviations

aCSF: artificial CSF
AD: Alzheimer’s disease
AMPA: α-amino-3-hydroxy-5-methylisoxazole-4-propionate
EPSC: excitatory post-synaptic current
LTP: long-term potentiation
NMDAR: NMDA receptor
*PS* cDKO: *presenilin* conditional double knockout mice
TBS: theta burst stimulation
